# Disposable optics for microscopy diagnostics

**DOI:** 10.1038/srep16957

**Published:** 2015-11-20

**Authors:** Pauliina Vilmi, Sami Varjo, Rafal Sliz, Jari Hannuksela, Tapio Fabritius

**Affiliations:** 1 Optoelectronics and Measurement Techniques Laboratory, Department of Electrical Engineering, University of Oulu, Finland; 2 The Center for Machine Vision Research, Department of Computer Science and Engineering, University of Oulu, Finland

## Abstract

The point-of-care testing (POCT) is having increasing role on modern health care systems due to a possibility to perform tests for patients conveniently and immediately. POCT includes lot of disposable devices because of the environment they are often used. For a disposable system to be reasonably utilized, it needs to be high in quality but low in price. Optics based POCT systems are interesting approach to be developed, and here we describe a low-cost fabrication process for microlens arrays for microscopy. Lens arrays having average lens diameter of 222 μm with 300 μm lens pitch were fabricated. The lenses were characterized to have standard deviation of 0.06 μm in height and 4.61 μm in diameter. The resolution limit of 3.9μm is demonstrated with real images, and the images were compared with ones made with glass and polycarbonate lens arrays. The image quality is at the same level than with the glass lenses and the manufacturing costs are very low, thus making them suitable for POCT applications.

Point-of-care testing (POCT) is medical testing at or near the location of a patient. This distinguishes it from traditional testing. Earlier all the samples were taken to a laboratory for testing, because of the need for large devices. With the development of the technology, smaller and portable equipment are now available; therefor the testing can be taken to the patient. POCT consists of fast and smart diagnostics devices which give immediate response and therefore test and therapeutic turnaround time is reduced. POCT is usually pleasant for the patient and because of its many benefits^1^ also doctors have an interest to use it more^2^. When the samples can be tested near the source, they are not transported and, for example tubs for blood collection are not needed anymore.

Because of the environment where the POCTs are utilized, many of the instruments and components need to be disposable, because sterilization is not always possible or practical. Disposable devices^3^ and small tests^4^ for POCT have been developed and demonstrated, and some of them do include optics, but still little focus is given to disposable optics itself.

One useful POCT device is a small portable microscope, which is very useful in health diagnostics in developing countries, where many globally important parasitic infections such as schistosomiasis or malaria are often under- or misdiagnosed. Conventional microscopy is good for this type of diagnostics, but it is expensive and the samples need to be taken to a laboratory for analysis, which causes unnecessary delay. Therefore there is a need for portable, robust and easy-to-use tools^5^.

These types of small microscopes require also small optics, and micro lens arrays (MLA) are perfect for this. Microlenses are also useful components for other optical applications, as they can be used for variety different purposes. For example, as single lenses they can be used to improve coupling to an optical fiber^6^, or as a lens array to improve luminance in a display^7^ and what is really interesting, to capture and display 3D-images, which can be observed without any special glasses^8^. They have also been used with conventional microscopes to capture light fields that enable new exiting imaging methods^9^.

The semi microscopic imaging, using microlens arrays as optics, has been shown to be a technique that allows capturing images of objects being as small as five micrometres in size. The typical egg sizes of parasites causing schistosomiasis range between 75 and 150 μm. An imaging technique has been demonstrated earlier to be capable of detecting such eggs in an automated manner using a mobile computing platform^10^. However, the microlens arrays enabling required imaging quality has been so far limited to expensive glass lenses. As a material, polymer is much more affordable than glass and therefore it is applied a lot in research of MLA fabrication.

Problems with MLA optics arise when examining for example stool samples, where many highly infective diseases and viruses can be found^11^. The risk of contamination of the instrumentation, and thus other samples, is always present^12^, and expensive MLAs can be difficult to keep clean and sterile. For this reason disposable lens arrays would be practical. When the fabrication costs are low enough, this is also a valid option. For a consumer, proper lens array made of glass costs about 300 €. In order to make lens matrix based POCT systems feasible, the target price for a disposable lens array should be less than 5 euros. With these prices disposability is a proper alternative for an awkward cleaning process which is not always practical when working outside proper facilities.

Many different fabrication methods for polymer micro lens arrays have been developed, such as special drop-on-demand devices^13^ and variety of different moulding techniques^14^,^15^,^16^. For imaging purposes the lens needs to be as smooth as possible for high-quality images. Hot embossing is a simple method for polymer lenses, but it requires high-quality mould for proper images increasing the fabrication costs and making it difficult to tune the lens properties. This type of issues do not exist when inkjet printing is used; the shape of the lens is formed by surface tension, and thus the lens surface is as smooth as the printed material because of the principle of minimum energy. And when photolithographic patterning is used on the substrate to define the wanted lens placement, the lens shape can be tuned more precisely compared to a plane drop-on-demand printing. In addition, production yield increases with the patterning.

In this work a process is presented for a low-cost inkjet printed micro lens array. Profile of the printed lenses is measured and the resolution is demonstrated with real images. The images are compared to ones made with a glass and a hot embossed polymer lens arrays.

## Materials and Methods

### Fabrication process for MLA

A glass substrate (AF32ECO from Schott), thickness of 125 μm is cleaned with acetone and isopropanol, after which a thin layer (4.5 μm) of negative photoresist (SU-8 3005) is spin coated with parameters presented in Table 1. A pattern is exposed onto the substrate with μPG101 micro pattern generator (Heidelberg Instruments). The pattern consists of 600 holes sized of 200 μm in diameter with 100 μm space between. Total area of the MLA is 8.9 mm × 5.9 mm. The exposed resist is heated on a hotplate for 1 min at 65 °C and 2 min at 95 °C after which it is developed in mr_Dev 600 Developer for one min and rinsed with IPA.

**Figure 1:**
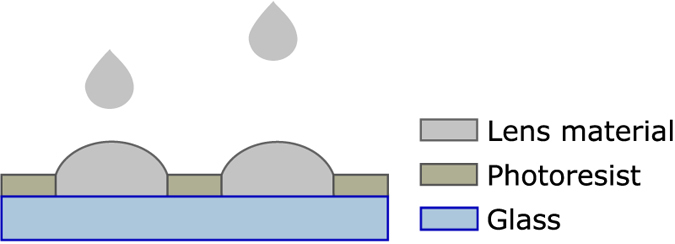
Inkjet printing of the lenses to the patterned substrate. The lenses are printed onto the patterned substrate drop by drop, until the shape is at wanted curvature.

**Figure 2:**
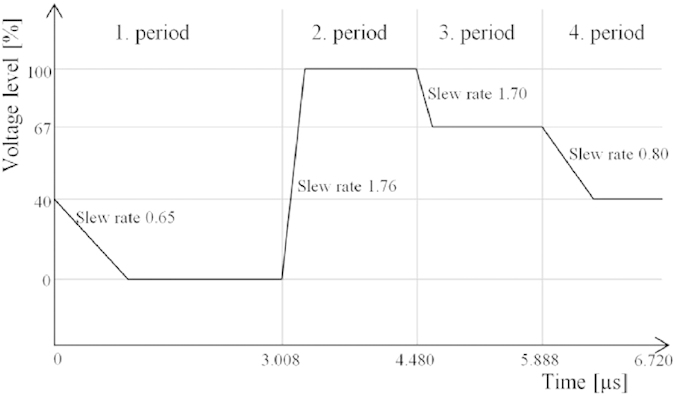
Printing settings for inkjet printing. The graph from the Dimatix material printer’s settings presents the voltage controlling the piezo-element, which pushes the droplet out of the nozzle. Voltage level 100% equals to chosen 24 V.

Inkjet printer is used to deposit the lenses into the holes of the resist layer. Figure 1 presents the idea of the process. The used material (InkEpo from Micro Resist Technology) is negative photoresist in liquid form, and is designed especially for optical purposes and is therefore highly transparent to visible light. The printer is drop-on-demand type (Dimatix 2800 series) and the droplet volume of the used cartridge is 10 pl. One nozzle is used and the settings for the nozzle are presented in Fig. 2. The nozzle voltage (equals Voltage level 100% in the Fig. 2) is 24 V. The “pools” are filled with 45 drops, drop by drop, until the liquid surface rises just above the resist layer edge to form a convex surface, a lens. For this application the radius of curvature for a lens is needed large, thus the lens is printed flat. After printing, the lenses are hardened with a normal procedure for photoresist, by pre-baking them at 85 °C for 10 min, then exposing at a wavelength of 365 nm for 10 s and finally baking at 95 °C for 5 min.

### Imaging system

**Figure 3:**
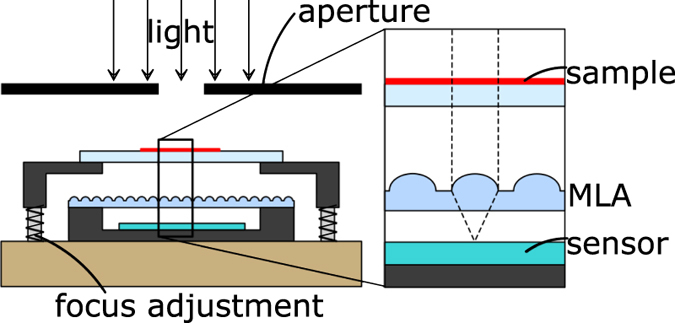
A conceptual image of the imaging setup. The system consists of a camera sensor, a microlens array, a sample holder and a light source with an aperture.

Direct imaging, using microlens arrays is a modified light field imaging technique that can be used for capturing images of semi microscopic targets^17^. Figure 3 illustrates the imaging concept. The system consists of a camera sensor, a microlens array, a sample holder and a light source with an aperture. The microlens array is positioned in front of the sensor so that each lens images the light source aperture without overlap on the camera sensor. The sample is positioned between the light source and the microlens array so that sharp images of the object are obtained.

The RAW images resemble the focused light field camera data^18^ and the image reconstruction can be done by the patch mosaicking approach or alternatively by the reparametrized back-projection^19^. The main advantages compared with conventional microscopes are the simplicity of the system and the scalability of the imaging area. While the system allows imaging target area of the camera sensor, the resolution is limited by Nyquist sampling theory and the sensor pixel size, as there is no magnification. However, the achieved resolution is useful for example for the detection of stained tumour cells or certain parasitic forms^10^.

## Results

For the finished MLA profile measurements are done and the shape of the lenses is studied. The imaging properties are tested with microscope targets.

### MLA characterization

**Figure 4:**
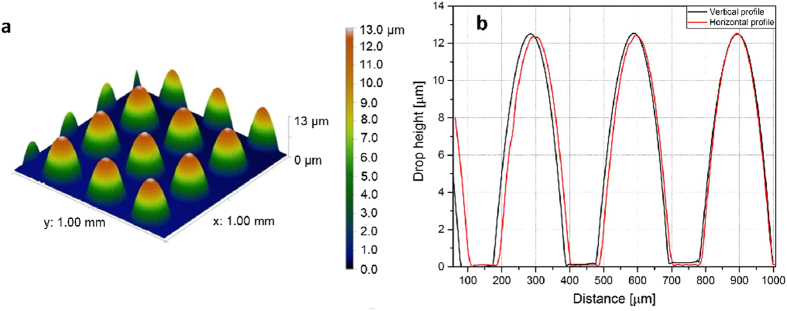
(**a**) 3D-profile of the printed MLA. (**b**) Graphs of the vertical and horizontal profiles of the MLA. The average height and diameter of the lenses is 12.4 μm with standard deviation of 0.06 μm and 222 μm with standard deviation of 4.61 μm, respectively.

Lens characterization is limited to profile measurements, which shows the uniformity of the lens array. Other properties (such as focal length) are not studied because of the demonstration-nature of this paper. The process steps still can be optimized, after which it would be more meaningful for thorough characterization. Profile measurement for the printed lenses is made with Veeco Dektak 8 surface profiler. Four areas of 1 mm × 1 mm were examined; the stylus scans the surface of the defined area and forms a 3D-image (Fig. 4a.). The shape of the lenses look uniform, and in Fig. 4b the vertical and horizontal profiles are presented together. All together 36 lenses are measured and average height is 12.4 μm with standard deviation of 0.06 μm. Average diameter is 222 μm with standard deviation of 4.61 μm. The small inconsistences in the curves are due to the Dektak’s feature of mathematically calculate the space between the measurement points. The graph also shows a small difference between the vertical and horizontal pitch of the lenses, because there has been a small distortion for the patterning when the pattern file is transformed in the lithography device. With different machinery this can be avoided.

The focal length was not measured due to lack of appropriate equipment, but from the measured dimensions of the lens, the approximate focal length was calculated from lensmaker’s equation, equation (1), which is simplified for plano-convex lens:


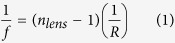



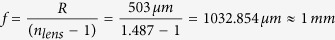


### Imaging by MLA

**Figure 5:**
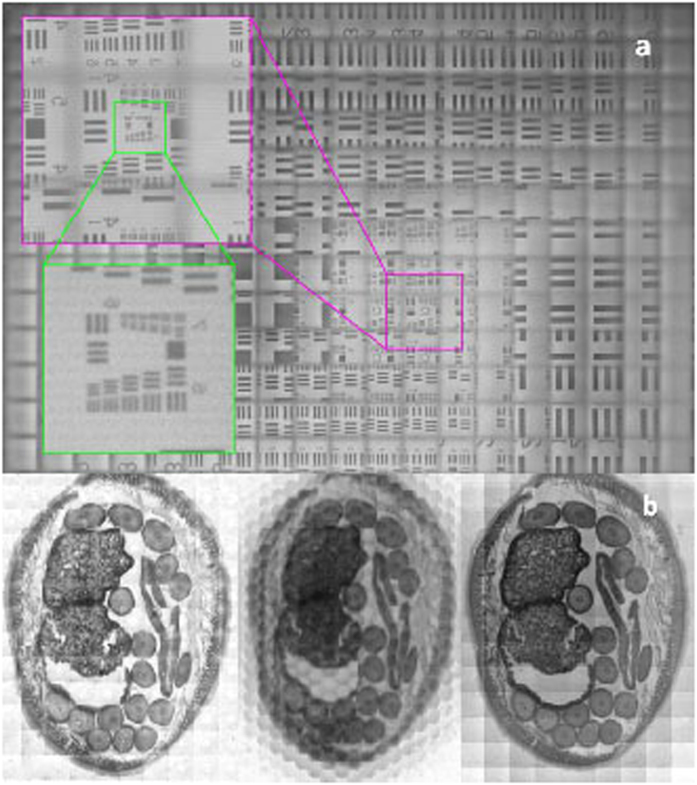
(**a)** Raw image, made with the printed lenses, showing zoomed area of the USAF resolution target demonstrating the limiting resolution of 3.9 μm. (**b)** Comparison of images made with different lens arrays. From left to right: glass lenses, hot embossed polycarbonate lenses and the proposed inkjet printed lenses. The polycarbonate lens array produces the lowest image quality. The inkjet lenses produce images with quality comparable to the class lenses.

Inkjet printed microlenses’ imaging properties are tested using microscope targets. The lighting is normalized by dividing the image data with an image of the light source aperture. Based on USAF resolution target (Edmun Optics, R209441-11069) the maximum obtainable resolution is 128 line pairs per millimetre or 3.9 μm line width (Fig. 5a). This is on the same level as demonstrated previously with glass microlenses^17^.

The reconstructed images of a roundworm (Ascaris) transverse section show clear improvement in an image quality with the inkjet manufactured lenses compared with the hot embossed polycarbonate lenses (Fig. 5b). Slight variation in light intensity can be observed in the result images. While this is not observed with the glass reference the overall image quality is at the same level than with the glass reference^17^.

## Discussion

The disposable optics has a great potential when considering the vast range of diagnostic and testing applications optics is utilized in. Many POCTs are based on chemical reactions, such as glucose meters^20^, which people with diabetes use. Optics based POCTs are needed when studying, for example tissue structures and cells, or when parasites in samples need to be identified. This can be done inside^21^ or outside of human body, thus disposable devices or parts are handy to prevent cross-contamination of samples and to maintain patient safety, even though many devices can be used over and over again. One more suitable application for these low-cost micro lenses could be in drug delivery, where the laser focused through the lenses creates small holes in the skin, which allow more effective absorption for the drug.

The presented process for MLAs is simple and easily tuneable for different sized and shaped lenses. Although, the inkjet printed lenses could be printed on a flat substrate with just a hydrophobic patterning, our process opens other possibilities for structures and shapes. Layers of different materials can be printed on top of each other with a high precision (such as different filters, like colour filter, with which colour images can be made), which is difficult for moulded lenses. Because of the patterning, the shape of the lens is not limited only to a circle, as with other drop-on-demand type processes. An ellipse, a polygonal or any shape is possible with the photolithography.

As a disposable component, the price is a key factor. For us the total price of one fabricated MLA rounded up approximately to 4 € excluding working hours. Fabrication in proper production line decreases the cost even more, when the materials are used more efficiently. The lenses are now printed on a glass substrate which, in sense on recycling, is challenging. The glass can be eliminated by printing the lenses on a PET substrate^22^, which would also decrease the prince of the MLA even more.

The width of the lenses is 20 μm more than the original lithography pattern made on the glass, because the material slightly spreads over the patterning, but this can be eliminated with a hydrophobic treatment on the patterning. It would make the shape of the lens even more easily tuneable, as there is only the height that is changing according to the amount of the printed material. In this demonstration, as the distance between the MLA and the sample is adjustable, the small change in the focal length, due to the spreading, is not significant.

This type of MLAs could also be applied in far-field imaging. The parameters and materials need to be changed, but the process itself would be the same. With thicker and non-transparent patterning, the lenses form image on the sensor without overlap.

The development of disposable optics is still at early stage, but because of all the positive effects it can have on point-of-care-testing, it is worth to give more focus to it in the future.

## Conclusion

Our study shows that low-cost fabrication method for micro lens array can produce lenses with quality of a same level than glass lenses. The height variation of the lenses is only 60 nm and the images produced with the lenses compete with those obtained with the glass reference lenses. Because of the low fabrication costs, the presented MLA is also a valid option to be used as a disposable optics component.

## Additional Information

**How to cite this article**: Vilmi, P. *et al.* Disposable optics for microscopy diagnostics. *Sci. Rep.* **5**, 16957; doi: 10.1038/srep16957 (2015).

## Figures and Tables

**Table 1:** Spin coating parameters.

	Speed	Total time	Ramp time
step 1	500 rpm	5 s	5 s
step 2	4000 rpm	60 s	5 s
step 3	1000 rpm	10 s	5 s
